# Regulating *p*-block metals in perovskite nanodots for efficient electrocatalytic water oxidation

**DOI:** 10.1038/s41467-017-01053-x

**Published:** 2017-10-16

**Authors:** Bo-Quan Li, Zi-Jing Xia, Bingsen Zhang, Cheng Tang, Hao-Fan Wang, Qiang Zhang

**Affiliations:** 1 Department of Chemical Engineering, Beijing Key Laboratory of Green Chemical Reaction Engineering and Technology, Tsinghua University, Beijing, 100084 China; 2 Shenyang National Laboratory for Material Science, Institute of Metal Research, Chinese Academy of Sciences, Shenyang, 110016 China

## Abstract

Water oxidation represents the core process of many sustainable energy systems, such as fuel cells, rechargeable metal-air batteries, and water splitting. Material surface defects with high-energy hanging bonds possess superb intrinsic reactivity, whose actual performance is limited by the dimension and conductivity of the electrocatalyst. Herein we propose a surface defect-rich perovskite electrocatalyst through a *p*-block metal regulation concept to achieve high performance for oxygen evolution. As a typical *p*-metal, Sn^4+^ dissolves from the solid phase from model SnNiFe perovskite nanodots, resulting in abundant surface defects with superior water oxidation performance. An oxygen pool model and a fusion-evolution mechanism are therefore proposed for the in-depth understanding of *p*-block metal regulation and the oxygen evolution reaction. The energy chemistry unveiled herein provides insights into water oxidation and helps to tackle critical issues in multi-electron oxygen electrocatalysis.

## Abstract (web summary)

Electrocatalysts that possess high densities of surface defects show great promise for efficient water oxidation. Here the authors demonstrate that regulating the *p*-block metal content in perovskite nanodots imparts these materials with abundant surface defects and excellent electrocatalytic activity.

## Introduction

Electrocatalysis constitutes the core process of abundant sustainable energy systems such as fuel cells, rechargeable metal-air batteries, and water splitting^1–3^. However, classical electrochemical reactions (for instance, oxygen evolution reaction (OER), oxygen reduction reaction (ORR), hydrogen evolution reaction (HER), etc.) are very sluggish in kinetics and strongly inhibit the efficiency of the energy devices^4–6^. Decades of researches have been carried out in exploring high-performance electrocatalysts, including noble metal compounds^7–9^, transition metal hydroxides^10–14^, oxides^15, 16^, perovskites^17–21^, sulfides^22–25^, metal-free nanocarbon^26–31^, and their composites^32–34^. Insights into the rational design of efficient electrocatalysts are strongly desired in this field in order to avoid tedious trial and error exploration.

The intrinsic reactivity, the amount of exposed active sites, and the electrical conductivity are the most significant aspects for a well-established electrocatalyst with excellent reactivity^35–37^. It is widely accepted that surface defects possess high intrinsic reactivity toward electrocatalysis^38–44^. The electrochemical reactions only happen at the surface of the electrocatalysts where feedstock/product can reach and electrons can be transferred during OER, ORR, and HER process. Surface defects with high-energy hanging bonds and dispersed empty electron orbitals are suitable for chemical adsorption and tend to interact strongly with reactants^43^.

Perovskites are a family of defect-abundant materials and exhibit excellent OER activity comparable to that of precious metal oxides^19, 45^. In a typical perovskite, A sites are usually alkaline earth metals, rare earth metals, or *p*-block metals while B sites are occupied with transition metals^46–49^. Both A sites and B sites are relatively easily substituted or dissociated^50, 51^, which usually results in an oxygen non-stoichiometry and thus oxygen deficiency. Such oxygen deficiency provides abundant surface defects, which are believed to be the active sites for OER^52–54^. Meanwhile, the concept of nano-structured electrocatalysts affords high surface area and abundant active sites exposed to feedstocks, and is therefore highly expected to promote the overall electrochemical performance^55–59^. A stable conductive framework is also strongly considered to guarantee full demonstration of the intrinsic reactivity of electrocatalysts by reducing the resistance of electron transportation not only at the reactive interface, but also between the electrocatalysts and the current collectors^37, 60^.

Based on this consideration, we introduced a unique strategy of *p*-block metal regulation of nano-sized perovskite electrocatalysts for OER. *P*-block metals possess a dual nature of metallicity and non-metallicity due to their unfilled *d* orbitals. In a certain alkaline condition, *p*-block metal ions precipitate to form crystallized hydroxides appearing as a typical metal element. When the condition changes through the rise of alkalinity or addition of extra ligands, the *p*-block metal ions dissociate from the solid phase as soluble coordination complexes because of their non-metallicity, resulting in abundant vacant sites at the surface. Utilizing such unique properties of *p*-block metals, we can construct in situ defects with high intrinsic reactivity at the surface of perovskite electrocatalysts.

## Results

### Material synthesis and characterization

**Fig. 1:**
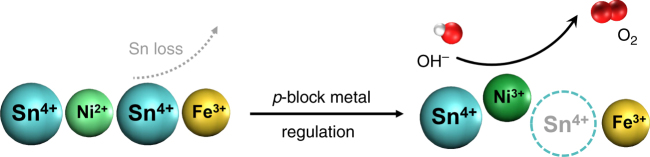
Schematic of *p*-block metal regulation of perovskite electrocatalysts for OER. Sn^4+^ dissociates from SnNiFe perovskite host and leaves in situ vacant sites during the *p*-block metal regulation. The surface vacant sites possess high intrinsic reactivity for water oxidation

To prove the concept, Sn is selected as a typical *p*-block metal to regulate SnNiFe perovskite hydroxide nanodots in situ hybridized with mesoporous graphene framework (MGF)^61^. Ni^2+^, Fe^3+^, and Sn^4+^ co-precipitate into crystalized perovskite hydroxides spatially confined within the mesopores of graphene, exhibiting the structure of SnNiFe perovskite nanodots strongly coupled with graphene framework. As illustrated in Fig. 1, Sn^4+^ dissociates from the perovskite host and leaves in situ vacant sites at the surface during the *p*-block metal regulated electrochemical activation. The loss of Sn^4+^ affords abundant surface defects with high reactivity and facilitates the oxidation of transition metal ions into higher oxidative states at the same time, which are considered as critical active species toward OER^62, 63^. The activated electrocatalyst exhibits efficient OER performance, superior to the state-of-art OER electrocatalyst IrO_2_ in aspects of reactivity, kinetics, and durability.

**Fig. 2:**
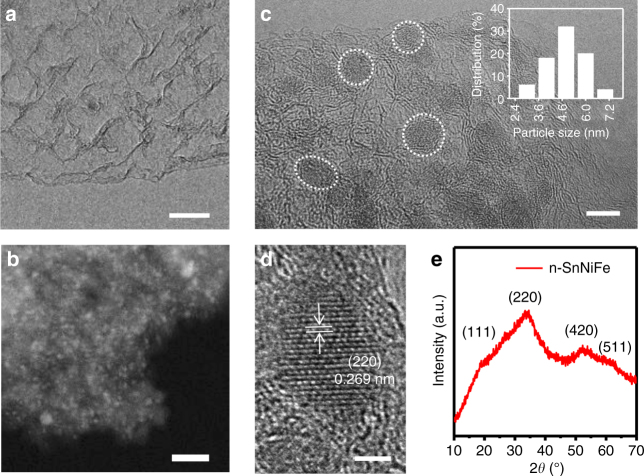
Morphology and structure characterization of perovskite nanodots. **a** TEM image of MGF, exhibiting a mesoporous graphene framework. **b** HAADF-STEM image and **c** TEM image of n-SnNiFe peroskite electrocatalyst. Inset figure in **c** is the size distribution of n-SnNiFe perovskite nanodots. **d** The high-resolution TEM image and **e** XRD patterns of perovskite nanodots spatially confined in mesoporous graphene of n-SnNiFe electrocatalyst. Scale bars, 10 nm (**a**), 5 nm (**b**), 20 nm (**c**), 2 nm (**d**)

The conductive MGF support was fabricated by a templated chemical vapor deposition (CVD) method. Scanning electron microscopy (SEM) and transmission electron microscopy (TEM) images demonstrate a hierarchical structure of MGF with plentiful mesopores with a uniform diameter within 10 nm (Fig. 2a; Supplementary Fig. 1). The hybrid electrocatalyst of SnNiFe perovskite hydroxide nanodots with MGF (named as n-SnNiFe) was synthesized by in situ co-precipitation. No bulk perovskite is observed at macro-scale morphology characterization (Supplementary Fig. 2). High angle annular dark field scanning TEM (HAADF-STEM) image in Fig. 2b exhibits the uniform distribution of perovskite nanodots in 3D graphene scaffolds. Further morphology characterization indicates the SnNiFe perovskite is spatially confined within the mesopores of MGF (Fig. 2c), resulting in SnNiFe nanodots with an average diameter of ca. 5 nm (Fig. 2c, inserted). The as-synthesized perovskite nanodots are well crystalized, which were proved by high-resolution TEM (Fig. 2d) and X-ray diffraction (XRD) patterns (Fig. 2e). The lattice fringes observed in Fig. 2d are identified to be (2 2 0), with the interplanar spacing being 0.269 nm.

Energy-disperse X-ray spectroscopy (EDS), X-ray photoelectron spectroscopy (XPS), and inductively coupled plasma optical emission spectrometer (ICP) were carried out for element analysis. The atomic ratio of Sn:(NiFe) of n-SnNiFe is close to 1 and Ni:Fe around 3.0 (Supplementary Fig. 3; Supplementary Table 1). The composition of n-SnNiFe is consistent with the theoretical stoichiometric ratio identified by comprehensive characterization. EDS mapping exhibits the uniform distribution of every element (Supplementary Fig. 4). The successful fabrication of n-SnNiFe perovskites attributes from the metallicity of transition metal and *p*-block metal. The n-SnNiFe with the structure of perovskite nanodots hybridized with conductive MGF is expected to fully demonstrate the intrinsic OER reactivity of the electrocatalyst.

### *P*-block metal activation and electrochemical evaluation

In order to create surface defects with intrinsic reactivity, *p*-block metal-regulated electrochemical activation of n-SnNiFe perovskite nanodots was performed. n-SnNiFe was electrochemically activated at a constant potential required to reach an initial current density of 5.0 mA cm^−2^ in O_2_-saturated 0.10 M KOH for 300 s. The *p*-block metal regulated n-SnNiFe electrocatalyst after electrochemical activation is named as p-SnNiFe.

**Fig. 3:**
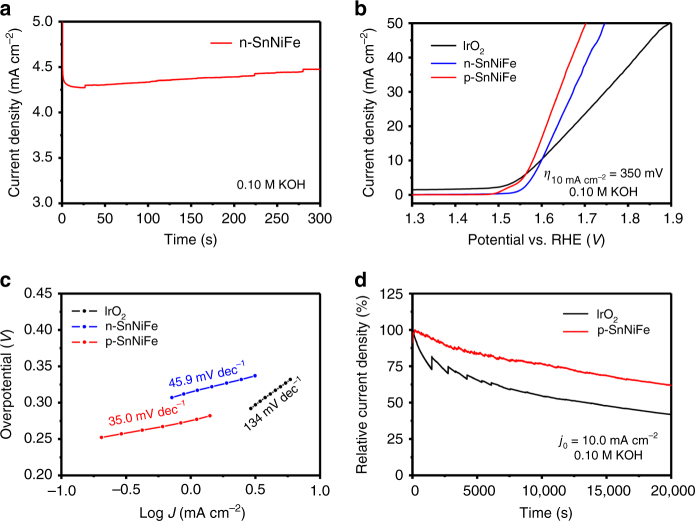
Electrochemical activation and characterization of perovskite electrocatalysts for OER. **a** Electrochemical activation of n-SnNiFe perovskite at a constant voltage required to reach an initial current density of 5.0 mA cm^−2^ in O_2_-saturated 0.10 M KOH. **b** 95% *iR*-compensated LSV profiles at a scan rate of 10.0 mV s^−1^ and **c** Tafel plots of n-SnNiFe, p-SnNiFe, and IrO_2_. **d** Chronoamperometric response at a constant potential required for an initial current density of 10.0 mA cm^−2^ of p-SnNiFe perovskite and IrO_2_

During the electrochemical activation, an obvious activation current was observed (Fig. 3a) with the current density raising from 4.3 to 4.5 mA cm^−2^. Figure 3b demonstrates the 95% *iR*-compensated linear sweep voltammetry (LSV) profiles at a scan rate of 10.0 mV s^−1^. The current density of p-NiFeSn perovskite electrocatalyst is distinctly increased compared with non-activated n-SnNiFe perovskite nanodots. The overpotential of p-SnNiFe at a current density of 10.0 mA cm^−2^ is 350 mV, which is among the best OER electrocatalysts (Supplementary Table 2) and even 20 mV better than IrO_2_, which is recognized as the state-of-the-art OER electrocatalyst.

Tafel plots (Fig. 3c) exhibit the superiority of p-SnNiFe perovskites (35.0 mV dec^−1^) over both n-SnNiFe perovskites (45.9 mV dec^−1^) and IrO_2_ (134 mV dec^−1^) with regard to OER kinetics. The stability of the electrocatalysts was characterized by a chronoamperometric method with an initial current density of 10.0 mA cm^−2^. The current density of p-SnNiFe retains 60% after 20,000 s test while only 40% of the current density of IrO_2_ is preserved, indicating better stability of p-SnNiFe perovskites against OER (Fig. 3d).

Bulk SnNiFe perovskite particles (named as b-SnNiFe) and the mechanical mixture of b-SnNiFe and MGF (named as b-SnNiFe + MGF) were prepared as control samples to demonstrate the significance of the unique pomegranate-like nanostructure of specially confined perovskites and highly conductive MGF toward both electrochemical activation and OER performance. The b-SnNiFe perovskite electrocatalyst exhibits a morphology of aggregated massive particles with an average diameter around 200 nm (Supplementary Fig. 5). The XRD patterns are identical with n-SnNiFe perovskites (Supplementary Fig. 6), along with similar stoichiometric ratio of Sn, Ni, and Fe (Supplementary Fig. 7; Supplementary Table 1) and element distribution (Supplementary Fig. 8), indicating the smart in situ hybridized design with MGF contributes to the fabrication of nano structures without interfering the nature of SnNiFe perovskites. Bulk SnNiFe perovskite particles share identical chemical constitution and structure with n-SnNiFe, and are only different in morphology of perovskite and existence of MGF, making ready for illustration of the necessity of pomegranate-like nanostructures with nano-sized perovskites and highly conductive graphene toward effective electrocatalysis.

The electrochemical activation process of b-SnNiFe and b-SnNiFe + MGF was similar to that of n-SnNiFe perovskites, and the *p*-block metal regulated samples after activation are named as pb-SnNiFe and pb-SnNiFe + MGF, respectively. The OER reactivity of b-SnNiFe and b-SnNiFe + MGF electrocatalysts was enhanced, which was suggested by the raised current density during in situ electrochemical activation and decreased overpotential (Supplementary Figs. 9, 10). For instance, the overpotential at 10.0 mA cm^−2^ was 20 mV decreased after electrochemical activation for b-SnNiFe + MGF. The improvement of OER reactivity indicates the universality of the *p*-block metal regulation strategy. However, the OER performance of neither pb-SnNiFe nor pb-SnNiFe + MGF is comparable with p-SnNiFe. The overpotential at 10.0 mA cm^−2^ of p-SnNiFe is 110 mV lower than that of pb-SnNiFe + MGF and far lower than pb-SnNiFe. In addition, the Tafel slope decreases from 190 mV dec^−1^ for pb-SnNiFe, 59.6 mV dec^−1^ for pb-SnNiFe + MGF to 35.0 mV dec^−1^ for p-SnNiFe (Supplementary Fig. 11). These evidences strongly confirm the superiority of p-SnNiFe in OER reactivity and kinetics.

In order to evaluate the stability of SnNiFe perovskite hydroxides, long-time durability tests were performed on b-SnNiFe. Carbon nanotube (CNT, with a mass ratio of 20%) was added to ensure the conductivity and the sample is named as b-SnNiFe + CNT. As is shown in Supplementary Fig. 12, the OER reactivity of b-SnNiFe + CNT electrocatalyst remains stable, with the overpotential at 10 mA cm^−2^ increases only 20 mV after 10,000 s, 25 mV after 20,000 s, and 42 mV after 40,000 s durability tests. The Tafel slopes exhibit similar tendency with slight increases. XRD patterns in Supplementary Fig. 13 and TEM images in Supplementary Fig. 14 further indicate the structure and morphology stability of perovskite hydroxides. The SnNiFe perovskite hydroxides exhibit satisfactory structural and electrochemical stability under OER condition in alkaline solution.

To further identify the origin of superiority of p-SnNiFe over pb-SnNiFe and pb-SnNiFe + MGF, electrochemical impedance spectroscopy (EIS) and electrochemical active surface area (ECSA) analysis were carried out. The electrical conductivity of nano-sized SnNiFe perovskites in situ hybridized with MGF is better for both pristine and electrochemical activated electrocatalysts (Supplementary Fig. 15). In addition, MGF contributes to the promotion of ECSA exhibited in Supplementary Fig. 16, which is characterized by the double-layer capacity. Considering the ability of electrochemical activation and OER performance, the nano-sized perovskite and conductive framework play a critical role to enhance the OER performance for SnNiFe perovskites. The rational design of nanostructure and hybridization with conductive framework is of great significance for a well-established electrocatalyst.

### Mechanism of *p*-block metal regulation

**Fig. 4:**
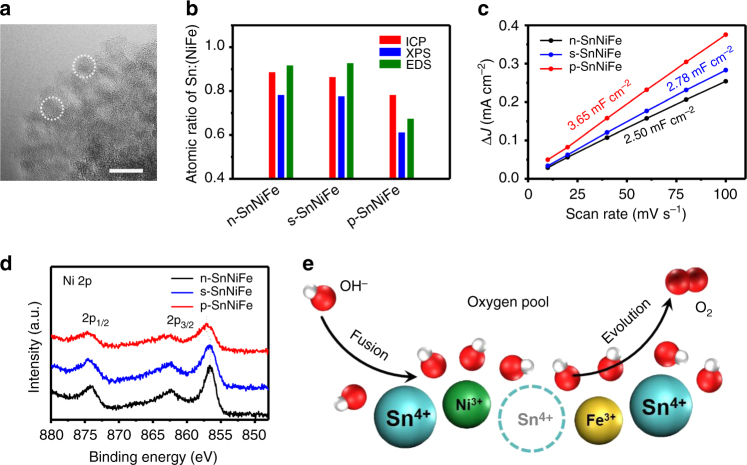
Evaluation of the *p*-block regulation strategy. **a** High-resolution TEM image of p-SnNiFe, exhibiting no obvious difference from n-SnNiFe. **b** Atomic ratio of Sn:(NiFe) of n-SnNiFe, s-SnNiFe, and p-SnNiFe determined by ICP, XPS, and EDS. **c** ECSA and **d** high-resolution Ni 2p XPS spectra of n-SnNiFe, s-SnNiFe, and p-SnNiFe. **e** Scheme of the proposed oxygen pool model and fusion-evolution OER process. Scale bars, 10 nm (**a**)

In order to verify the surface defects attributed to Sn loss and understand the mechanism of *p*-block metal regulation of n-SnNiFe perovskites, detailed characterization of activated p-SnNiFe perovskites was carried out. The s-SnNiFe perovskite was prepared by soaking n-SnNiFe perovskite in the electrolyte as a control sample. There is no significant change between p-SnNiFe and n-SnNiFe in either morphology or crystal structure as shown in Fig. 4a, Supplementary Figs. 17, 18, suggesting morphology is not the key factor leading to OER activation. In addition, neither the electrical conductivity nor the surface hydrophility of the electrocatalysts is relevant confirmed by EIS in Supplementary Fig. 19 and Supplementary Table 3 and contact angel in Supplementary Fig. 20, respectively. However, element analysis exhibits consistent results of Sn loss by EDS, XPS, and ICP analysis (Supplementary Figs. 21, 22, and Supplementary Table 1). The atomic ratio of Sn:(NiFe) decreases from ~0.9 of n-SnNiFe and s-SnNiFe to ~0.7 of p-SnNiFe (Fig. 4b), with the atomic ratio of Ni:Fe remains relative stable around 3 (Supplementary Fig. 23). The dissociation of Sn lefts abundant vacant sites, which is confirmed by enhanced ECSA of p-SnNiFe measured by capacitive current caused by adsorption and desorption of electrolyte (Fig. 4c).

Meanwhile, with the loss of positively charged Sn^4+^, transition metal ions in solid perovskite phase feel a stronger positive electric field. The transition metal ions tend to loss electron and therefore be oxidized to higher oxidative states. As shown in Fig. 4d, the high-resolution Ni 2p XPS spectra of p-SnNiFe exhibit a 0.60 eV shift to higher binding energy compared with n-SnNiFe and s-SnNiFe perovskites sharing similar Ni 2p XPS spectra. Such results strongly indicate the oxidation process and higher oxidative state of Ni in p-SnNiFe perovskites, which is considered to contribute to the enhanced OER performance. In contrast, the oxidative states of Fe^3+^ and Sn^4+^ remain relative stable during the activation as illustrated in Supplementary Fig. 24 by XPS Fe 2p spectra and Sn 3p spectra. Above all, the unique dual properties of metallicity and non-metallicity of Sn is the main factor for construction of surface defects and oxidation of active transition metal ions, resulting in improved electrocatalysts and excellent OER performance.

## Discussion

According to the discussion above, an oxygen pool model and a fusion-evolution mechanism are inferred and proposed for in-depth understanding of OER process. When Sn^4+^ dissolves during electrochemical activation, vacant sites are in situ created. Instead of staying at the original sites, lattice oxygen at the surface of the perovskite nanodots tends to form an amorphous layer with a more incompact oxygen arrangement, which is defined as the oxygen pool (Fig. 4e). Impelled by transition metal ions in high oxidative states, hydroxyl not only adsorbs on the surface of the electrocatalyst, but also fuses into the amorphous oxygen pool, identical to other hydroxyl in the amorphous solid state. The oxygen pool becomes crowded and excited with extra hydroxyl addition, which actuates electron transfer and product evolution, thus catalyzes the water oxidation. After the evolution of oxygen, the oxygen pool returns incompact and peaceful, and ready for another cycle of OER catalysis. Notably, such mechanism suggests the oxygen involved in product is not necessarily to be the same oxygen from the feedstock, but possibly comes from the lattice oxygen in a certain degree^64, 65^. The oxygen pool model and the fusion-evolution process ingeniously explain the electrochemical activation and further inspire us for deeper understanding of OER and rational design of electrocatalysts.

In conclusion, we proposed the strategy of *p*-block metal regulation for rational design of electrocatalysts using the unique dual nature of metallicity and non-metallicity of *p*-block metal. The proof-of-concept SnNiFe perovskite nanodots spatially confined within MGF were fabricated. During the *p*-block metal regulated electrochemical activation, Sn^4+^ dissolves from solid phase, resulting in abundant surface defects with high intrinsic reactivity and transition metal ions oxidized to higher oxidative states favorable for OER catalysis. The activated perovskite electrocatalyst exhibits excellent OER performances, with an overpotential required for 10.0 mA cm^−2^ to be 350 mV in alkaline electrolyte, which is comparable to the state-of-art IrO_2_ electrocatalyst. The creative oxygen pool model is proposed and describes that the amorphous oxygen surface after electrochemical activation is more inclined to reactant fusion and product evolution. Both the *p*-block metal regulation strategy and the oxygen pool model inspire us for rational electrocatalyst design and in-depth understanding of electrocatalysis. Therefore, the strategy of *p*-block metal regulation reported herein not only proves to be effective and rational for constructing defect-rich highly active electrocatalysts, but also enlightens in-depth understanding of mechanism of electrocatalysis.

## Methods

### Synthesis of MGF

MGF was prepared through a CVD method using mesoporous MgO as template and CH_4_/NH_3_ as carbon/nitrogen source, respectively. The MgO template was synthesized by a facile hydrothermal reaction using commercial MgO. Typically, 2.20 g polyethylene glycol (PEG)-2000 was dissolved in 100 mL deionized water and 1.0 g MgO was added. The slurry was stirred for 48 h and then transferred into a 200 mL Teflon autoclave for hydrothermal reaction at 200 °C for 48 h. The precipitate was filtered, washed with deionized water for three times and dried at 80 °C overnight. The mesoporous MgO template was obtained after calcination of the as-obtained precursor at 650 °C for 5.0 h. The CVD growth was carried out in a furnace with MgO template placed in the middle of a horizontal quartz tube. The reactor was heated to 950 °C under Ar flow (150 mL min^−1^) with a heating rate of 20 °C min^−1^. Both CH_4_ (70 mL min^−1^) and NH_3_ (30 mL min^−1^) were simultaneously introduced into the reactor for 15 min after the temperature was stable, and then the furnace was cooled naturally to room temperature under Ar protection. The product was purified using 6.0 mol L^−1^ HCl at 95 °C for 12 h to fully remove the MgO template. After filtering, washing with deionized water and ethanol, and freeze-drying for 24 h, MGF was obtained for further synthesis.

### Synthesis of perovskite electrocatalysts

n-SnNiFe was synthesized by in situ co-precipitation. About 100 mg MGF was dispersed into 30 mL N-methylpyrrolidone (NMP) under sonication for 30 min to form a homogeneous suspension. About 1.5 mmol Ni(NO_3_)_2_·6H_2_O, 0.50 mmol Fe(NO_3_)_3_·9H_2_O, and 2.0 mmol citric acid were dissolved in 50 mL deionized water under stirring for 10 min and then mixed with the MGF slurry. About 10 mL SnCl_4_ aqueous solution (0.2 mol L^−1^) was added into the above mixture under vigorous stirring. After that, 1.0 mol L^−1^ NaOH was added dropwise into the as-obtained solution under sonication until the pH reached 10. The sonication went for another 30 min to accelerate the precipitation reaction. The product was separated by centrifuging at 10,000 rpm for 10 min and purified by washing with deionized water and ethanol for three times, respectively. The n-SnNiFe was finally obtained after freeze-drying for 24 h. b-SnNiFe was synthesized under otherwise identical conditions without MGF. b-SnNiFe + MGF was prepared by simply mixing b-SnNiFe and MGF under grind.

### Electrochemical activation of perovskite electrocatalysts

The electrochemical activation process was carried out using a three-electrode system. The samples for activation were prepared using the same method as the working electrode fabrication for electrochemical measurements. The activation conditions were identical with electrochemical evaluation process, with the electrolyte to be O_2_-saturated 0.10 M KOH (see more details in the electrocatalysis section). All samples were electrochemically activated at a constant potential required to reach a specific initial current density for 300 s. n-SnNiFe and b-SnNiFe + MGF were activated at the initial current density of 5 mA cm^−2^, resulting in p-SnNiFe and pb-SnNiFe + MGF, respectively. The initial current density of b-SnNiFe was determined to be 2.5 mA cm^−2^ due to its poor reactivity and the final product was pb-SnNiFe. s-SnNiFe was prepared as control sample by simply soaking n-SnNiFe into the electrolyte for 300 s.

### Characterization

The morphology was characterized using a JSM 7401F (JEOL Ltd., Tokyo, Japan) SEM at 3.0 kV and a JEM 2010 (JEOL Ltd.) TEM at 120.0 kV. EDS analysis and mapping were performed using the JEM 2010 TEM equipped with an Oxford Instrument energy dispersive X-ray spectrometer. High-resolution transmission electron microscope (HRTEM) images were recorded using a FEI Tecnai G^2^ F20 TEM. XRD patterns were collected at 40.0 kV and 120 mA with Cu-K_α_ radiation on Bruker D8 Advanced Diffractometer. XPS measurements were carried out by Escalab 250xi, with all XPS spectra corrected using C 1s line at 284.6 eV. Elemental analysis was performed using an inductively coupled plasma optical emission spectrometer (IRIS Intrepid II XSP, ThermoFisher, USA). The contact angle was measured using an OCAH200 Optical contact angle measuring instrument (Dataphysics, Germany).

### Electrocatalysis

Electrochemical measurements were performed using a three-electrode system controlled by a CHI 760D electrochemistry station (CH Instrument, USA). A platinum sheet electrode served as the counter electrode. A saturated calomel electrode (SCE) was used as the reference electrode. The working electrode was a rotating disk electrode with a disk diameter of 5.0 mm.

The working electrode was fabricated using the following method: 5.0 mg electrocatalyst was dispersed in 0.95 mL ethanol and 0.05 mL Nafion solution (5.0 wt%) and then sonicated for 30 min to form a homogeneous suspension. In total, 10.0 μL suspension was dropped onto the glass carbon disk electrode, which was polished in advance. The working electrode was ready for electrochemical measurements or activation after the solvent was evaporated. The areal loading mass of the working electrode was 0.25 mg cm^−2^. The working electrode of electrochemical activated perovskites was directly used for further electrochemical measurements.

All electrochemical measurements were carried out in O_2_-saturated 0.10 mol L^−1^ KOH electrolyte at room temperature. The working electrode was rotated at 1600 rpm during the tests. All potentials measured were corrected to reversible hydrogen electrode (RHE) using the following equation: *E*_RHE_ = *E*_SCE_ + 0.241 V + 0.0592 pH.

OER performance of the electrocatalysts was evaluated by LSV at a scan rate of 10.0 mV s^−1^. All polarization profiles were corrected with 95% *iR*-compensation. Tafel slopes were calculated based on LSV curves using the Tafel equation *η* = *b* log(*j/j*_*0*_). *η* is the overpotential calculated using the following equation: *η* = *E*_RHE_−1.23 V, *b* is the Tafel slope, *j* is the current density, and *j*_*0*_ is the exchange current density. ECSA was evaluated by the double-layer capacitance (*C*_dl_), which was determined by capacitive current dependent on the scan rate of cyclic voltammetry (CV). The CV measurements were carried out at a scan window from 0.000 to 0.050 V vs. SCE with scan rates of 10, 20, 40, 60, 80, and 100 mV s^−1^. The double-layer capacitance was half of the slope calculated by fitting the Δ*j* = *j*_*a*_−*j*_*b*_ at 0.025 V vs. SCE against the scan rate, which is used to represent the ECSA. The EIS was carried out at 0.57 V vs. SCE over a frequency range from 0.10 to 100 kHz at the sinusoidal voltage amplitude of 5.0 mV. The stability test of the electrocatalysts was characterized at a constant voltage required to reach an initial current density of 10.0 mA cm^−2^.

### Data availability

All data generated or analysed during this study are included in this published article (and its Supplementary Information files).

## Electronic supplementary material

Supplementary Information
