# Supplementary material for: Regulating p-block metals in perovskite nanodots for efficient electrocatalytic water oxidation
Source: Nat Commun. 2017 Oct 16;8:934. doi: 10.1038/s41467-017-01053-x (PMC5643308; doi:10.1038/s41467-017-01053-x)
Supplement: Supplementary file 1 — Supplementary Information [file 41467_2017_1053_MOESM1_ESM.pdf]

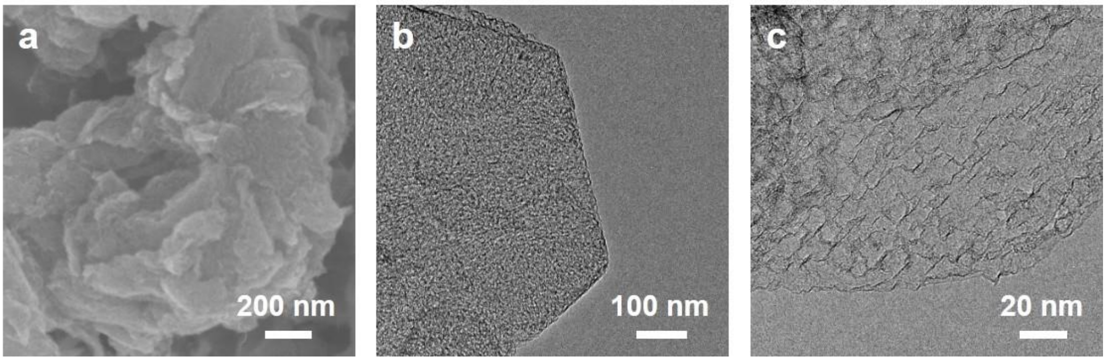

Supplementary Figure 1: Morphology of MGF. (a) SEM image, (b) & (c) TEM images of MGF, exhibiting graphene nanosheets with abundant mesopores.

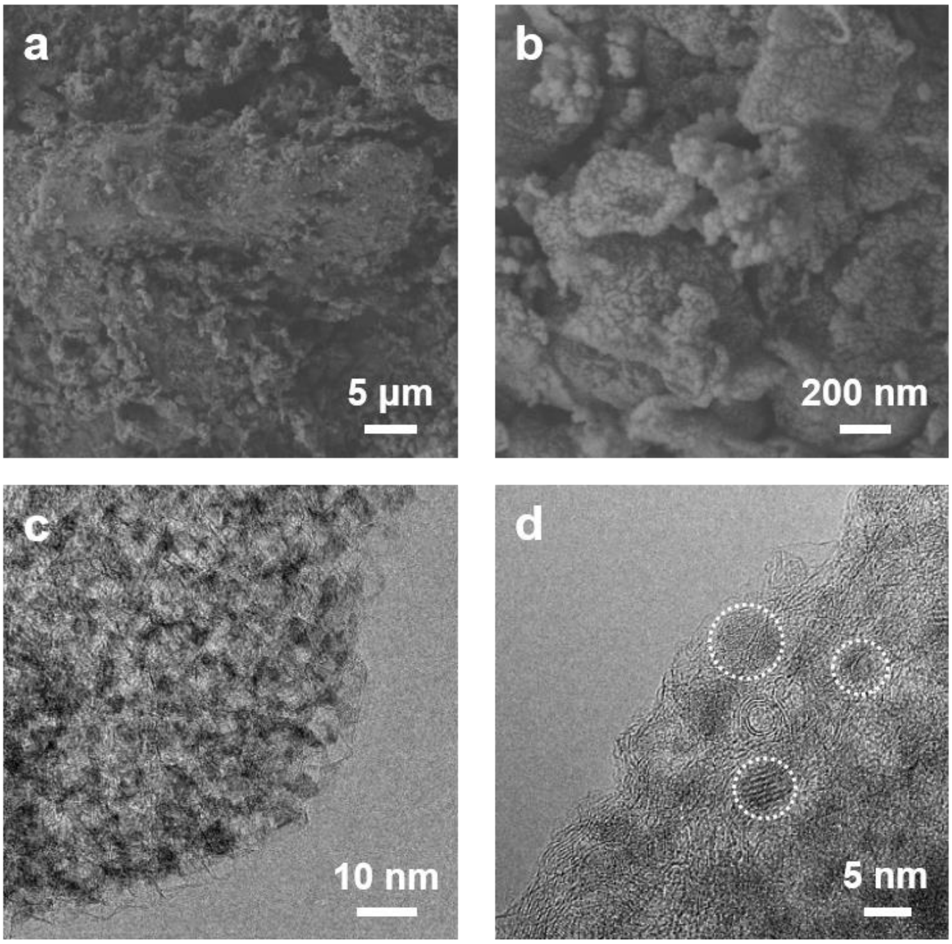

Supplementary Figure 2: Morphology of n-SnNiFe perovskite nanodots. (a) & (b) SEM images and (c) & (d) TEM images of n-SnNiFe. Perovskite nanodots are specially confined within the mesopores of MGF.

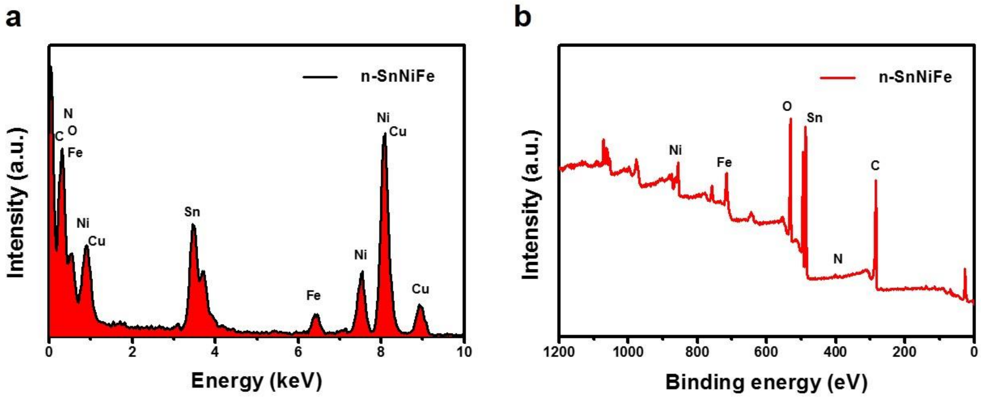

Supplementary Figure 3: Elemental composition analysis of n-SnNiFe perovskite. (a) EDS pattern and (b) XPS survey spectrum of n-SnNiFe.

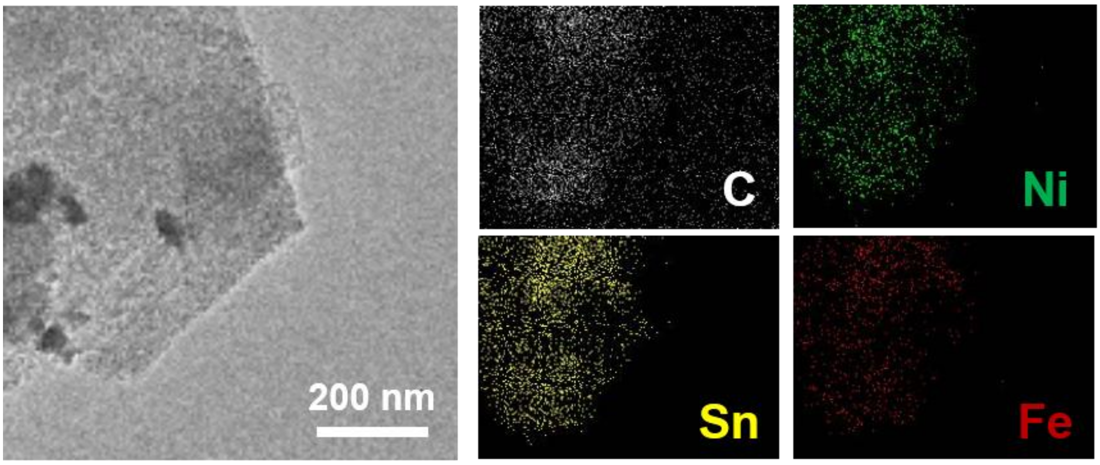

Supplementary Figure 4: EDS mapping of n-SnNiFe electrocatalyst, exhibiting a uniform distribution of elements of SnNiFe perovskite nanodots at MGF.

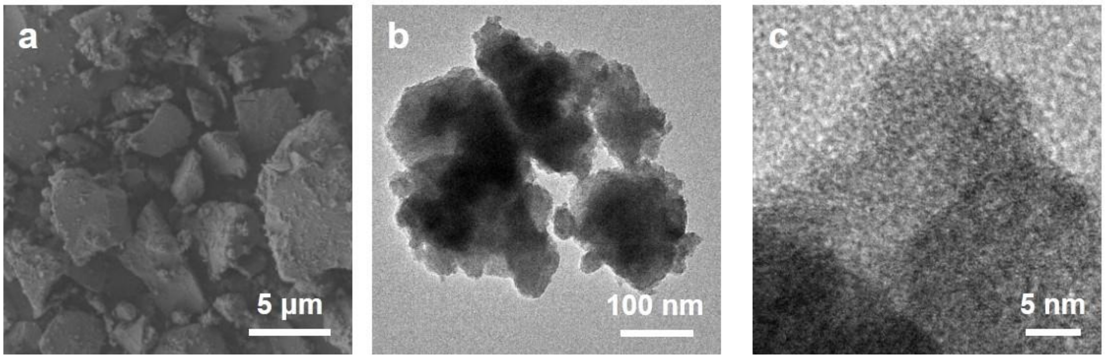

Supplementary Figure 5: Morphology of b-SnNiFe perovskite particles. (a) SEM image and (b) & (c) TEM images of b-SnNiFe, exhibiting bulk SnNiFe perovskite particles with an average diameter of 200 nm.

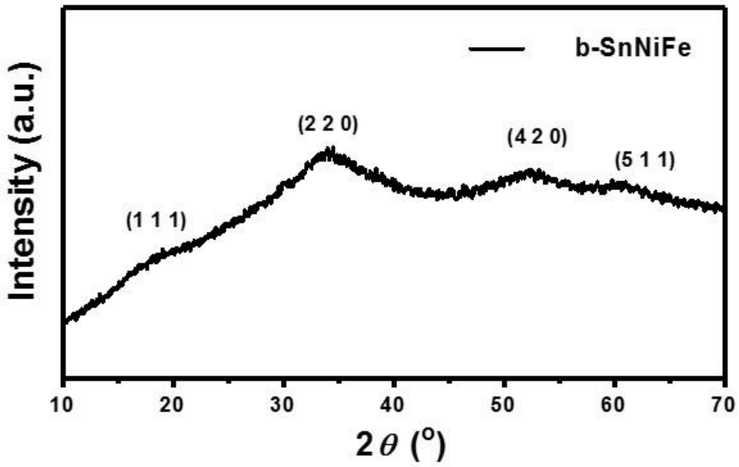

Supplementary Figure 6: XRD patterns of b-SnNiFe perovskite. The XRD patterns are similar to that of n-SnNiFe, suggesting identical crystal structure with n-SnNiFe perovskite.

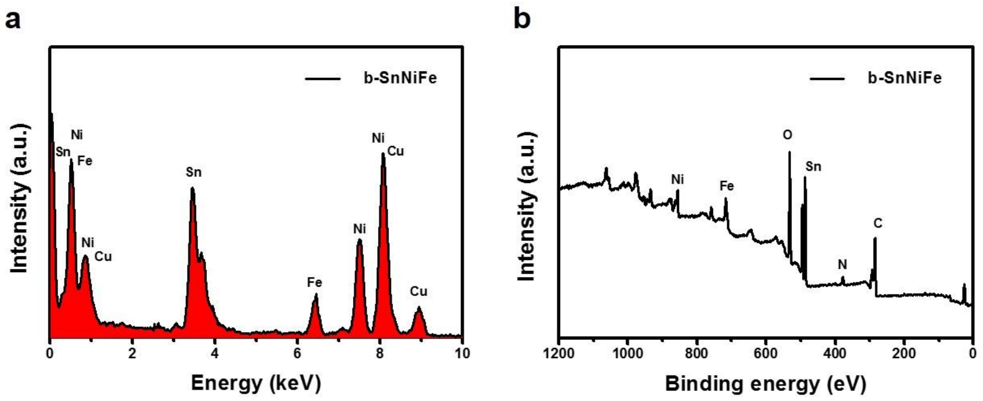

Supplementary Figure 7: Elemental composition analysis of b-SnNiFe perovskite. (a) EDS pattern and (b) XPS survey spectrum of b-SnNiFe perovskite.

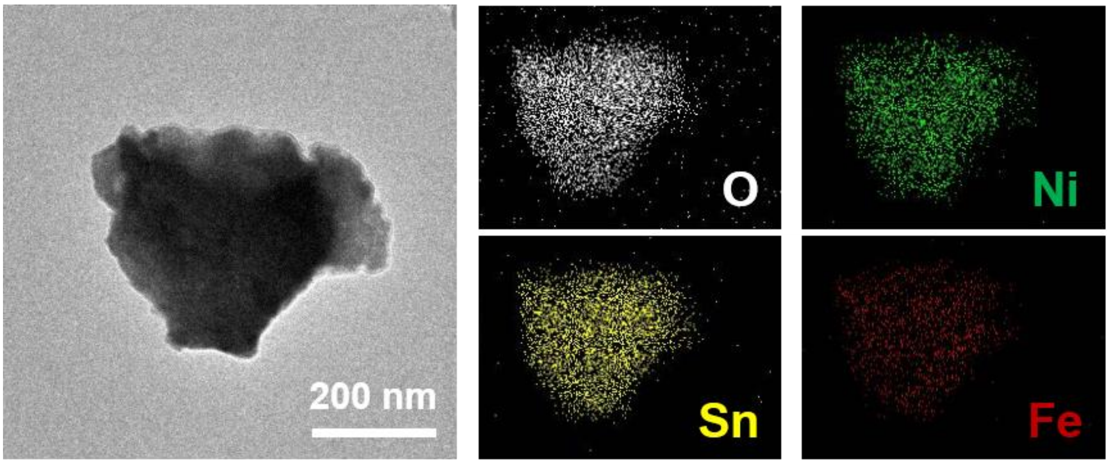

Supplementary Figure 8: EDS mapping of b-SnNiFe perovskite electrocatalyst. All elements distribute uniformly in b-SnNiFe electrocatayst.

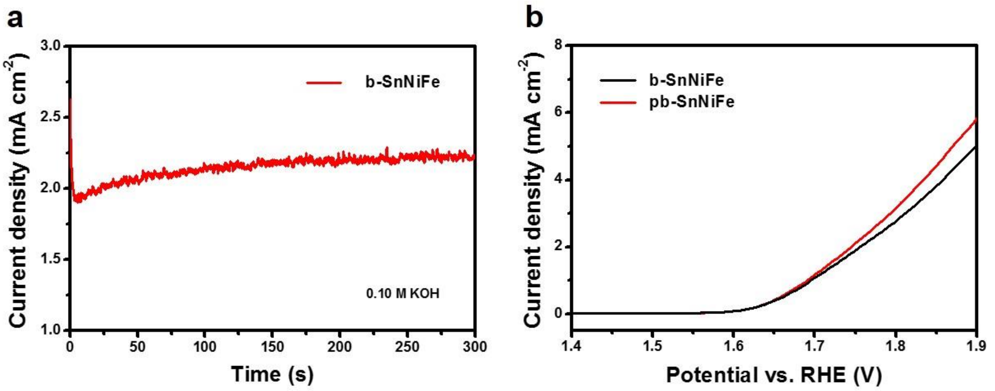

Supplementary Figure 9: Electrochemical activation of b-SnNiFe perovskite in O_2-saturated 0.10 M KOH. (a) Electrochemical activation of b-SnNiFe perovskite at a constant potential required to reach an initial current density of 2.5 mA cm^{-2}. (b) 95% *iR*-compensated LSV profiles at a scan rate of 10.0 mV s^{-1} of b-SnNiFe and pb-SnNiFe. The activation is not as obvious as n-SnNiFe perovskite.

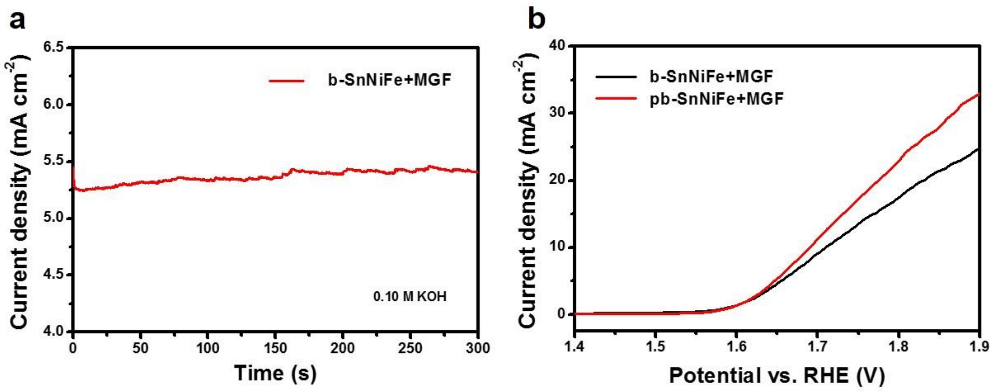

Supplementary Figure 10: Electrochemical activation of b-SnNiFe+MGF in O_2-saturated 0.10 M KOH. (a) Electrochemical activation of b-SnNiFe+MGF at a constant potential required to reach an initial current density of 5.0 mA cm^{-2}. (b) 95% *iR*-compensated LSV profiles at a scan rate of 10.0 mV s^{-1} of b-SnNiFe+MGF and pb-SnNiFe+MGF. The activation effect is stronger than b-SnNiFe perovskite but still inferior to n-SnNiFe perovskite.

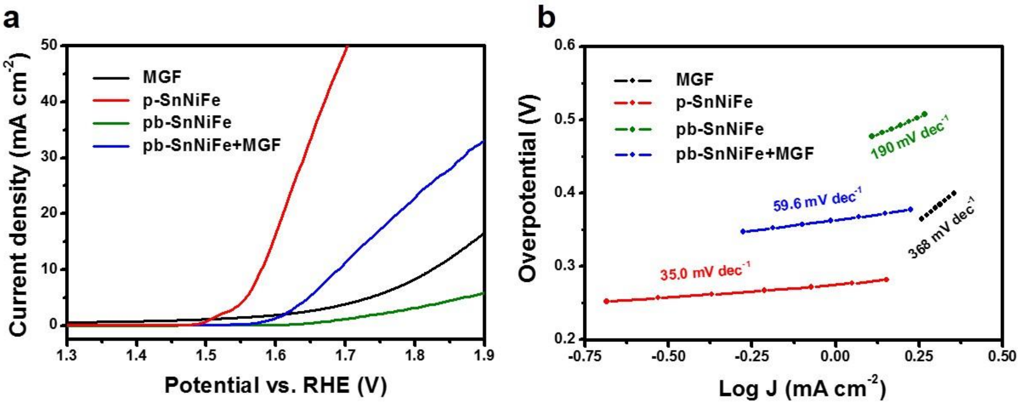

Supplementary Figure 11: OER performance characterization of electrochemical activated electrocatalysts in O_2-saturated 0.10 M KOH. (a) 95% *iR*-compensated LSV profiles at a scan rate of 10.0 mV s^{-1} and (b) Tafel plots of MGF, p-SnNiFe, pb-SnNiFe, and pb-SnNiFe+MGF. p-SnNiFe perovskite exhibits superior OER performance among all electrocatalysts in this work.

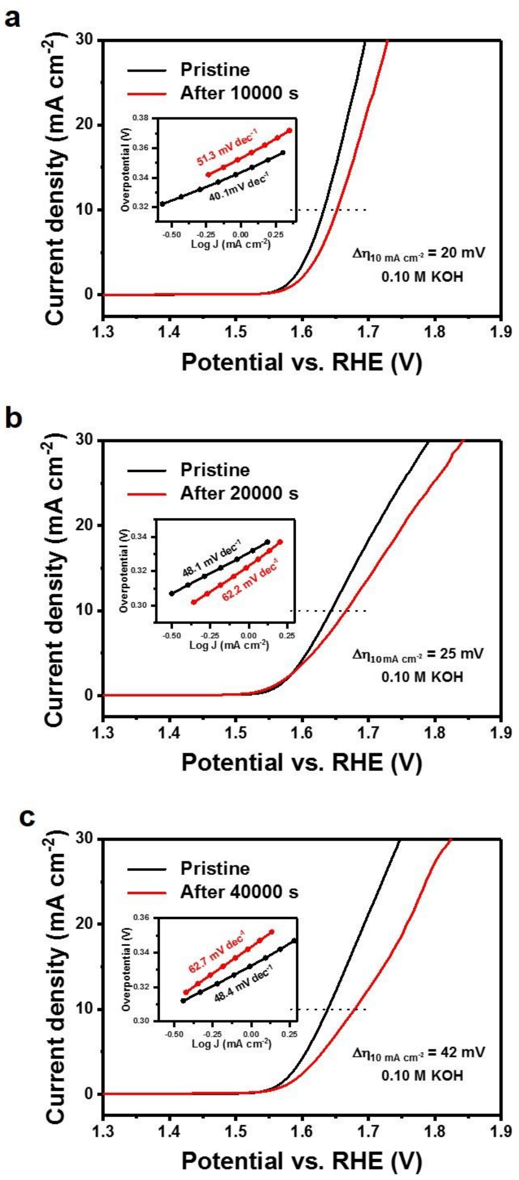

Supplementary Figure 12: Stability characterization of b-SnNiFe+CNT electrocatalysts. The b-SnNiFe+CNT electrocatalysts were tested using chronoamperometric method 0.10 M KOH solution at a constant potential to reach an initial current density of 10.0 mA cm^{-2}. 95% *iR*-compensated LSV profiles at a scan rate of 10.0 mV s^{-1} and inserted Tafel plots of b-SnNiFe+CNT electrocatalysts after (a) 10000 s, (b) 20000 s, and (c) 40000 s durability tests.

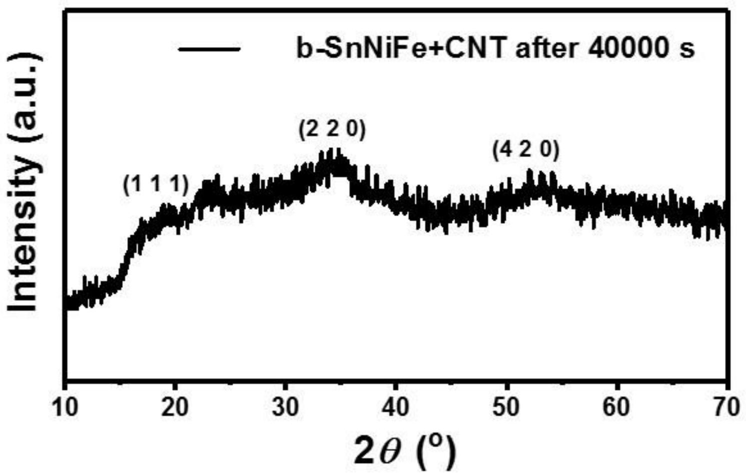

Supplementary Figure 13: XRD patterns of b-SnNiFe+CNT electrocatalyst after 40000 s durability test. The XRD patters are similar to the pristine b-SnNiFe electrocatalyst, indicating the structural stability of perovskite hydroxides under OER conditions.

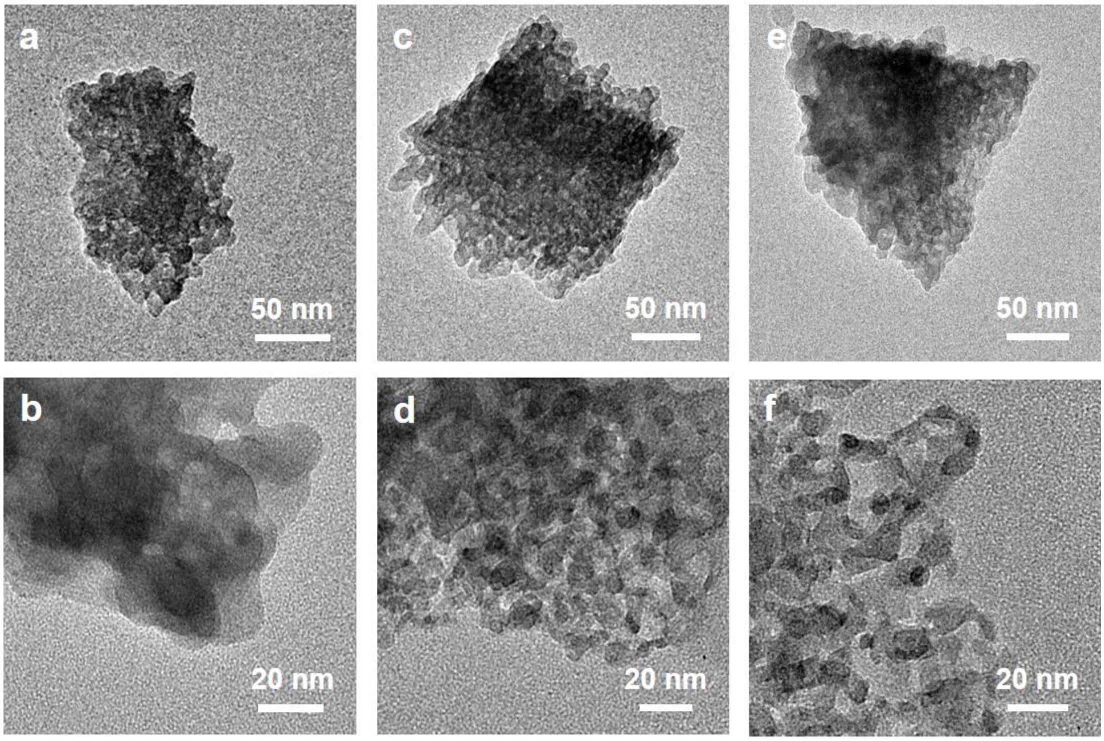

Supplementary Figure 14: Morphology of b-SnNiFe+CNT electrocatalysts after durability tests. TEM images of b-SnNiFe+CNT electrocatalysts after (a) & (b) 10000 s, (c) & (d) 20000 s, and (e) & (f) 40000 s stability tests under OER conditions in 0.10 M KOH solution.

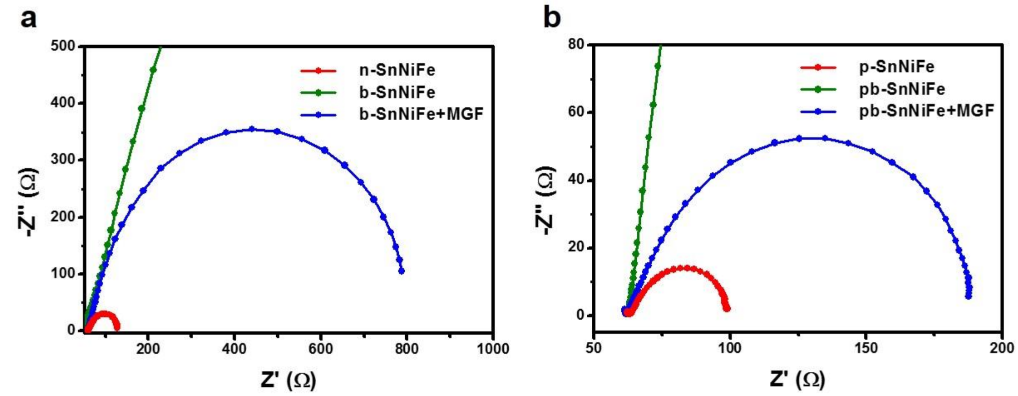

Supplementary Figure S15: EIS characterization of n-SnNiFe, b-SnNiFe, b-SnNiFe+MGF, p-SnNiFe, pb-SnNiFe, and pb-SnNiFe+MGF. Nano-structured SnNiFe perovskite exhibits the lowest resistance for both pristine and electrochemical activated electrocatalysts. The addition of MGF also contributes to conductivity improvement.

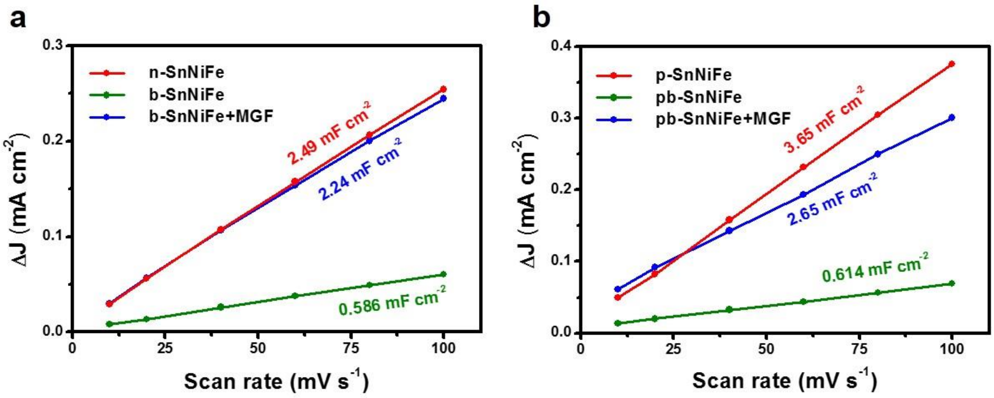

Supplementary Figure 16: ECSA characterization of n-SnNiFe, b-SnNiFe, b-SnNiFe+MGF, p-SnNiFe, pb-SnNiFe, and pb-SnNiFe+MGF. p-SnNiFe perovskite exhibits the highest double-layer capacity, indicating the largest ECSA among all electrocatalysts in this work.

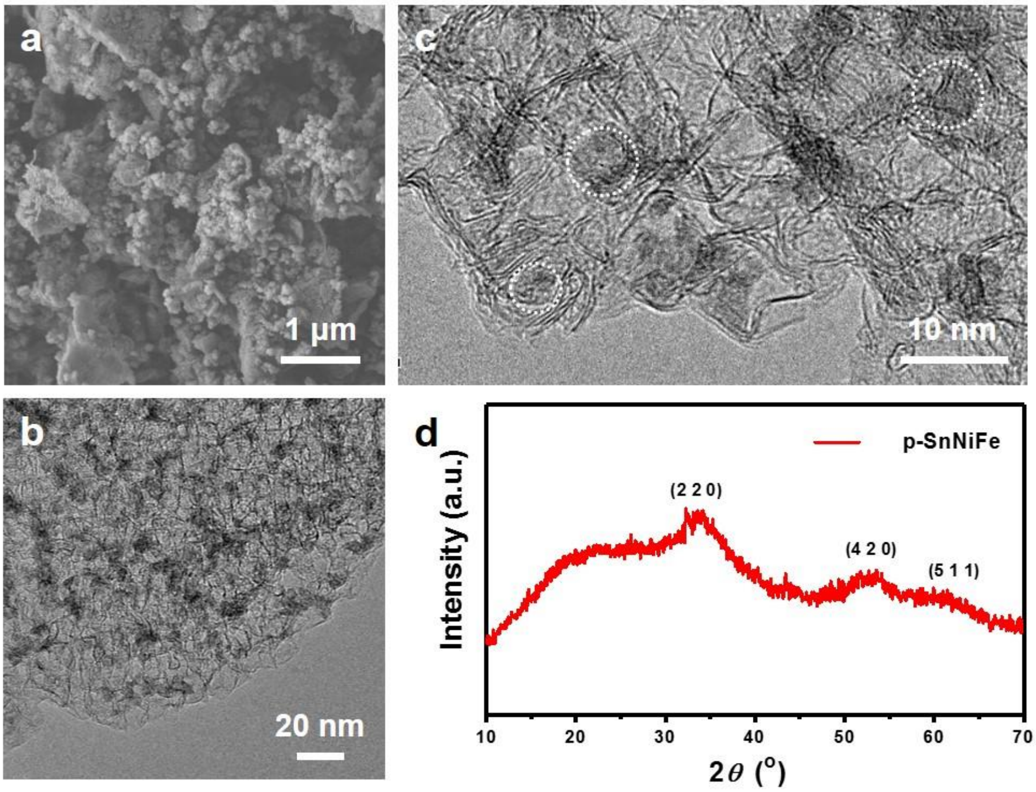

Supplementary Figure 17: Morphology and structure characterization of p-SnNiFe perovskites. (a) SEM image, (b) TEM image, (c) high-resolution TEM image, and (d) XRD patterns of p-SnNiFe perovskite, demonstrating no significant difference from n-SnNiFe in aspect of morphology or crystal structure.

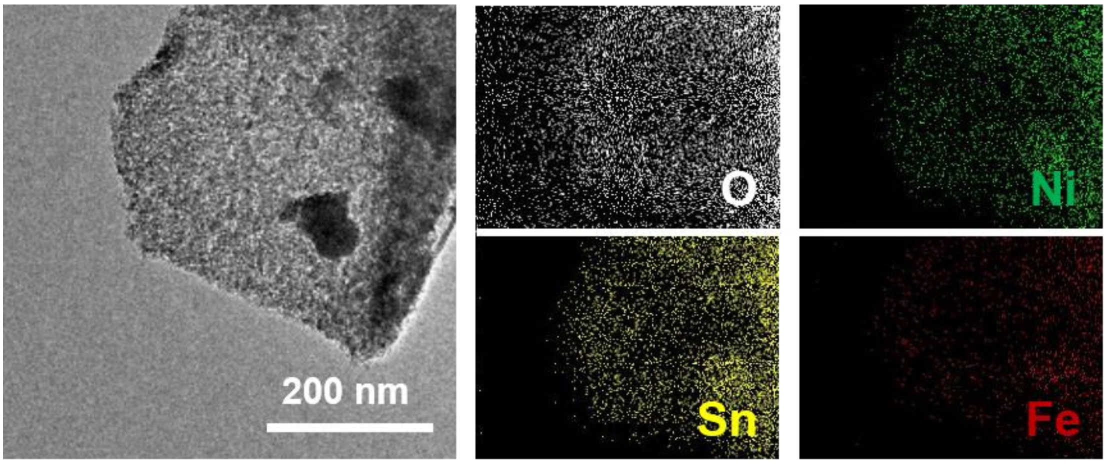

Supplementary Figure 18: EDS mapping of p-SnNiFe perovskite electrocatalyst. No obvious aggregation of any element is observed.

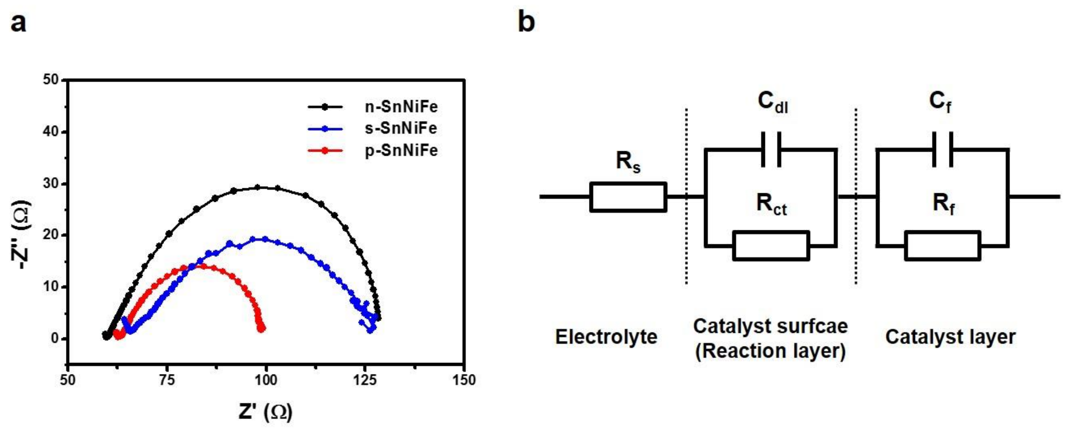

Supplementary Figure 19: EIS analysis of n-SnNiFe, s-SnNiFe, and p-SnNiFe electrocatalysts. (a) EIS spectra of the electrocatalysts. (b) Equivalent circuit used to fit the impendance data in (a). R_s is the resistance of the electrolyte, R_{ct} is the charge transfer resistance, and R_f is the resistance of catalyst layer. C_{dl} is the double layer capacitance and C_f is the capacitance of catalyst layer. The results demonstrate similar resistance among the electrocatalysts, indicating electric conductivity is not the key factor for improvement of OER performance during electrochemical activation.

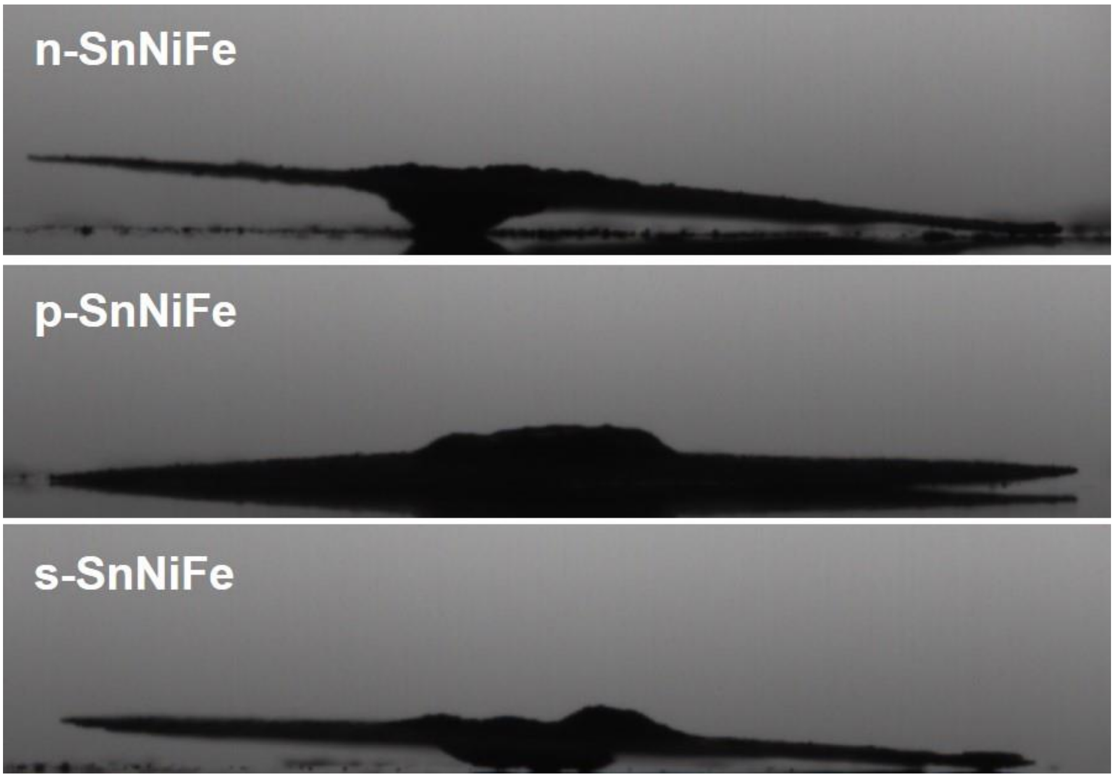

Supplementary Figure 20: Contact angle of n-SnNiFe, p-SnNiFe, and s-SnNiFe electrocatalysts. For all the samples, the water drop was instantly adsorbed and no obvious contact angle is detected, exhibiting high surface hydrophility of all the electrocatalysts.

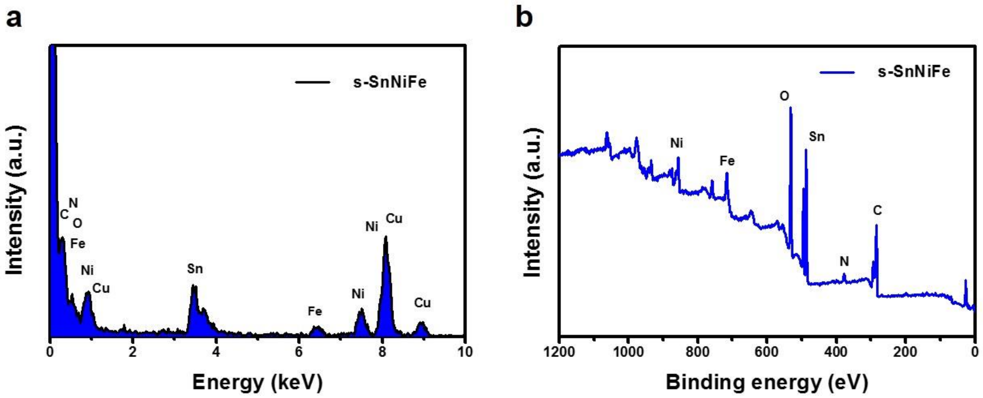

Supplementary Figure 21: Elemental composition analysis of s-SnNiFe perovskite. (a) EDS pattern and (b) XPS survey spectrum of s-SnNiFe perovskite.

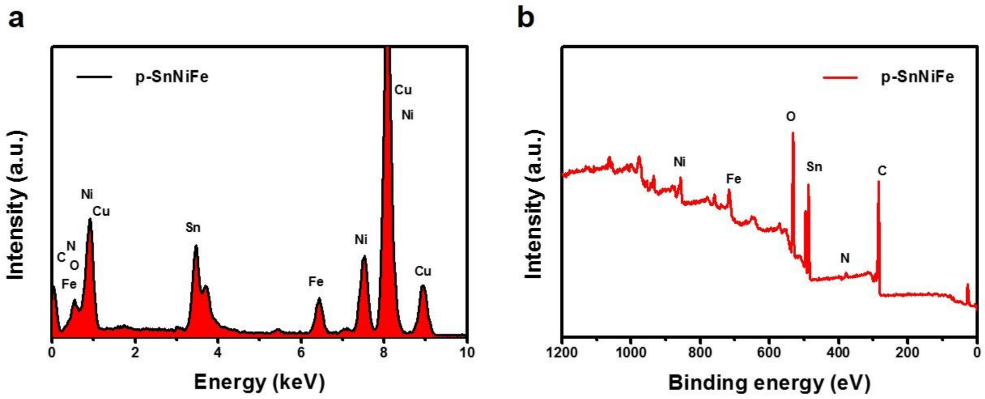

Supplementary Figure 22: Elemental composition analysis of p-SnNiFe perovskite. (a) EDS pattern and (b) XPS survey spectrum of p-SnNiFe perovskite.

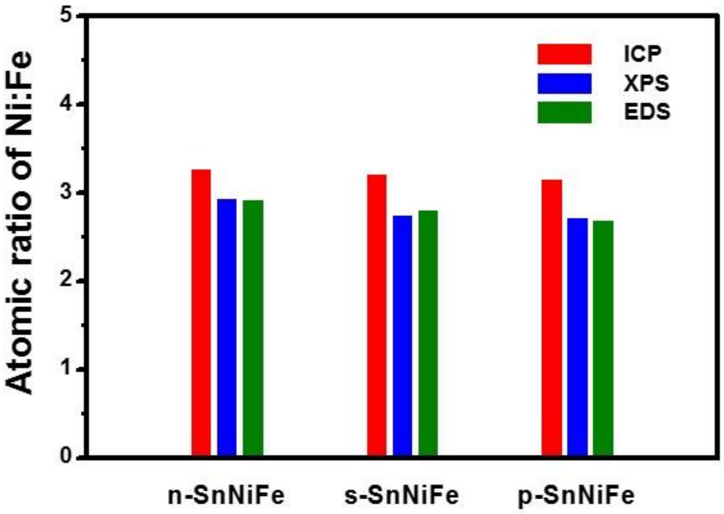

Supplementary Figure 23: Atomic ratio of Ni:Fe of n-SnNiFe, s-SnNiFe, and p-SnNiFe electrocatalysts characterized by ICP, XPS, and EDS. No obvious difference of the atomic ratio of Ni:Fe among the samples is observed, indicating the composition of Ni and Fe is relative stable.

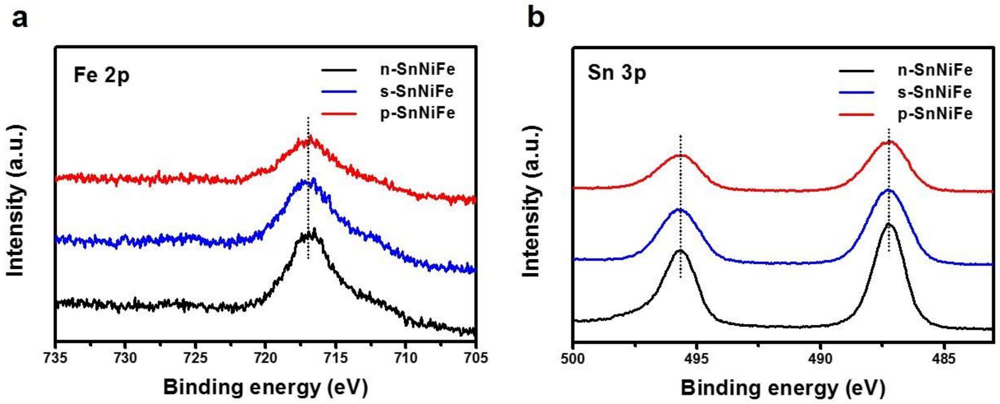

Supplementary Figure 24: High-resolution (a) Fe 2p XPS spectra and (b) Sn 3p XPS spectra of n-SnNiFe, s-SnNiFe, and p-SnNiFe perovskite. No obvious shift is observed, indicating the oxidative state of Fe^{3+} and Sn^{4+} stayed relative stable during the electrochemical activation.

Supplementary Table 1: Summary of electrocatalyst compositions

Sample	Method	Relative amounts of elements (at. %)			Atomic ratio of Sn:(NiFe)	Atomic ratio of Ni:Fe
		Sn	Ni	Fe		
b-SnNiFe	ICP	43.6	37.2	11.4	0.895	3.257
	XPS	7.7	7.2	2.5	0.794	2.86
	EDS	18.5	15.2	5.6	0.862	2.73
n-SnNiFe	ICP	12.2	10.6	3.2	0.886	3.255
	XPS	3.7	3.5	1.2	0.782	2.93
	EDS	5.6	4.6	1.6	0.917	2.91
s-SnNiFe	ICP	11.3	9.9	3.1	0.864	3.197
	XPS	4.0	3.8	1.4	0.776	2.74
	EDS	6.2	4.9	1.8	0.927	2.80
p-SnNiFe	ICP	9.2	8.95	2.9	0.782	3.137
	XPS	2.4	2.9	1.1	0.612	2.70
	EDS	10.0	10.8	4.0	0.674	2.68

Supplementary Table 2: OER performance of electrocatalysts for comparison.

Samples	η@10 mA cm^{-2} (mV)	Electrolyte	Working electrode	Loading mass (mg cm^{-2})	Reference
n-SnNiFe	370				
p-SnNiFe	350	0.10 M KOH	RDE	0.25	This work
IrO_2	370				
n-NiFe LDH@NGF	337	0.10 M KOH	RDE	0.25	1
$ZnCo_2O_4$/N-CNT	430	0.10 M KOH	RDE	0.20	2
N-Co_9S_8/G	409	0.10 M KOH	RDE	0.20	3
$SrNb_{0.1}Co_{0.7}Fe_{0.2}O_{3-\delta}$	420	0.10 M KOH	RDE	0.25	4
LSCF-OCNT	403	0.1 M KOH	RDE	0.21	5
$Co_3O_4/NiCo_2O_4$	340	1.0 M KOH	NF	1.0	6
Ni–Co mixed oxide cages	380	1.0 M KOH	RDE	-	7
SCP	480	0.10 M KOH	RDE	0.232	8

Supplementary Table 3: Quantitative analysis of the resistance parameters of electrocatalysts corresponding to EIS spectra.

Sample	R_s (Ω)	R_{ct} (Ω)	R_f (Ω)
n-SnNiFe	60.32	6.79	56.98
p-SnNiFe	63.34	5.62	32.47
s-SnNiFe	66.36	8.92	44.39

Supplementary references

1. Zhu, X. *et al.* Dual-sized NiFe layered double hydroxides in situ grown on oxygen-decorated self-dispersal nanocarbon as enhanced water oxidation catalysts. *J. Mater. Chem. A* **3**, 24540 −24546 (2015).
2. Liu, Z.−Q., *et al.* $ZnCo_2O_4$ quantum dots anchored on nitrogen−doped carbon nanotubes as reversible oxygen reduction/evolution electrocatalysts. *Adv. Mater.* **28**, 3777−3784 (2016).
3. Dou, S., Tao, L., Huo, J., Wang, S. & Dai, L. Etched and doped Co_9S_8/graphene hybrid for oxygen electrocatalysis. *Energy Environ. Sci.* **9**, 1320−1326 (2016).
4. Zhu, Y. *et al.* $SrNb_{0.1}Co_{0.7}Fe_{0.2}O_{3-\delta}$ perovskite as a next−generation electrocatalyst for oxygen evolution in alkaline solution. *Angew. Chem. Int. Ed.* **54**, 3897−3901 (2015).
5. Elumeeva, K. *et al.* A simple approach towards high−performance perovskite−based bifunctional oxygen electrocatalysts. *ChemElectroChem* **3**, 138−143 (2016).
6. Hu, H., Guan, B., Xia, B. & Lou, X. W. Designed formation of Co_3O_4/$NiCo_2O_4$ double−shelled nanocages with enhanced pseudocapacitive and electrocatalytic properties. *J. Am. Chem. Soc.* **137**, 5590−5595 (2015).
7. Zhang, B. *et al.* Homogeneously dispersed multimetal oxygen−evolving catalysts. *Science* **352**, 333−337 (2016).
8. Zhu, Y., *et al.* Phosphorus−doped perovskite oxide as highly efficient water oxidation electrocatalyst in alkaline solution. *Adv. Funct. Mater.* **26**, 5862−5872

(2016).
